# COVID-19 Life Events Spill-Over on Family Functioning and Adolescent Adjustment

**DOI:** 10.1177/02724316211036744

**Published:** 2022-03

**Authors:** Andrea M. Hussong, Allegra J. Midgette, Adrianna N. Richards, Rachel C. Petrie, Jennifer L. Coffman, Taylor E. Thomas

**Affiliations:** 1 2331 University of North Carolina at Chapel Hill, Chapel Hill, NC, USA; 2 14616 University of North Carolina at Greensboro, Greensboro, NC, USA

**Keywords:** parent-adolescent relationships, stress, communication, family

## Abstract

We examined US parent and youth perceptions of how life events, both positive and negative, associated with COVID-19 resulted in changes in family and youth functioning. Families (*n* = 105, 80% white, 48% male, and 87% mothers) completed surveys during the pandemic (May to July 2020) and 3 years prior (for youth ages *M = 10.6, SD = 1.17 and M = 13.6, SD = 1.19)*. Declines in youth, though not parent, report of open family communication, parental support, and family satisfaction were found. Declines were associated with various domains of pandemic-related stress in parent report, though positive life events served as buffers. Pre-pandemic family functioning also predicted pandemic stress. Spillover effects in turn impacted youth functioning. The current findings shed light on how experiences of the pandemic are linked with family functioning and have implications for how to support families during this time.

The COVID-19 pandemic and associated public and governmental responses have led to rapid and at times drastic changes in the life of the family (e.g., Chung et al., 2020b; Craig & Churchill, 2020; Gambin et al., 2020). Although some of these changes result in stressors that challenge family functioning (Chung et al., 2020a; Fontanesi et al., 2020) others may offer opportunities to preserve or even bolster family functioning in the face of the pandemic (Orgilés et al., 2020). Emerging research from around the world documents the impact of COVID-19 and previous epidemics/pandemics on families in terms of economic and resource loss, work-related stress, isolation and separation, illness and exposure concerns, caregiving burdens, and schooling needs for children (Ammar et al., 2020; Brooks et al., 2020; Brown et al., 2020; Chung et al., 2020b; Craig & Churchill, 2020; Del Boca et al., 2020; Garcia de Avila et al., 2020; Hawryluck et al., 2004; Orgilés et al., 2020; Saurabh & Ranjan, 2020; Segre et al., 2020). Evidence that these stressors impact the mental and physical health of both parents and children is growing (Brown et al., 2020; Del Boca et al., 2020; Orgilés et al., 2020; Patrick et al., 2020; Saurabh & Ranjan, 2020; Segre et al., 2020; Xie et al., 2020). However, still unclear are how such stressors impact family systems, the factors that preserve family functioning during the pandemic, and the links between family functioning and youth mental health during this time. We address these issues in a longitudinal study of families who have youth in early to mid-adolescence and who reside primarily in the southeastern United States with an emphasis on indices of family climate (i.e., satisfaction), process (i.e., communication), and salient dyadic relationship interactions (i.e., parental support of youth).

## Pandemic Life and Family Stress

**Figure 1.**
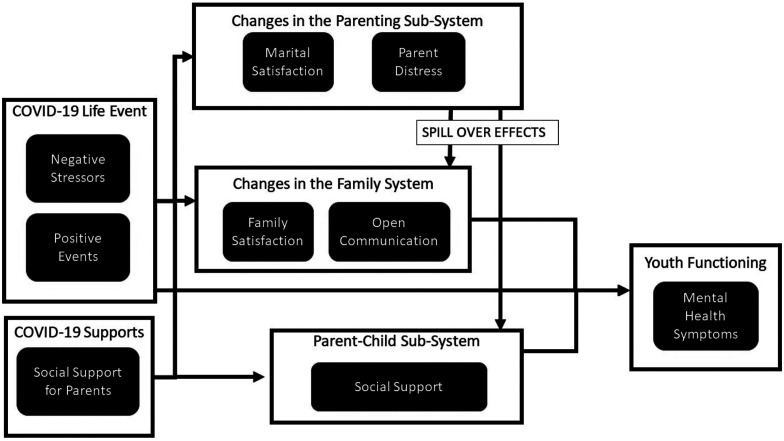
Conceptual model.

Family Stress Models (Price et al., 2016) suggest that one way in which the pandemic may be impacting family functioning is through negative life events that create greater family strain and disruption, leading to decreased family satisfaction as reported by individual family members as well as to reductions in open and effective family communication (see conceptual model in Figure 1). In addition, such negative life events may directly strain the parent–child sub-system and lower the extent to which youth feel supported by parents. Whether impacting the broader family system or the parent–child sub-system directly, negative stressors may lead to increased mental health symptoms associated with the pandemic in youth by increasing strain on family systems.

Spillover models (Kouros et al., 2014) suggest that pandemic-related negative life events may also have indirect effects on youth functioning. Such negative life events, including changes to parents’ employment, financial strain, and family caretaking, increase parents’ own distress as well as the support and satisfaction they experience in their marital relationships. For example, Kouros et al. (2014) found that how parents respond to stress spills over into their relationships within the family such that aspects of family functioning (e.g., marital relationships and parenting behaviors) in turn impact children’s adjustment. In addition to those impacting parents directly, pandemic stressors for parents may also include how children cope with their own negative life events, such as changes in schools and isolation related to the pandemic. Parenting stress models extend spillover models by suggesting that the ways in which children manage stress can impact family systems, including by evoking parenting behaviors that shape family relationships, family conflict, and parental functioning (Zemp et al., 2017).

Together, family stress, spillover, and parenting stress models posit that stressors for any one family member impact the functioning of the family system more generally, with implications for youth mental health. With the far-reaching life events linked with the COVID-19 pandemic (Brooks et al., 2020), the impact of stressors may be amplified by simultaneous direct effects on each family member and the family system at large as well as by indirect influences of these stressors to strain family relationships and functioning. In this way, pandemic stressors may echo in the family, creating cycles of disruption in family processes and relationships that impact individual adjustment.

As one key example of a broad pandemic stressor, families around the world report being affected by quarantine and mandatory social distancing (Brooks et al., 2020). These quarantine practices result in challenges to work–family balance through less support for parents from employers and spouses (Chung et al., 2020b; Craig & Churchill, 2020), unemployment (Brown et al., 2020), online schooling demands (Segre et al., 2020), and increased caregiver burden (Russell et al., 2020; Patrick et al., 2020). Although quarantine may entail spending more time at home with family, Ammar et al. (2020) found that family members in Asia, Africa, and Europe reported feeling more socially isolated because of fewer opportunities to visit with family outside of the home as well as friends and neighbors during quarantine.

There is mounting evidence that such pandemic stressors are associated with a host of changes in individual functioning. COVID-19-related changes have been associated with greater parental stress as well as mental health symptoms including anxiety and depression (Chung et al., 2020b; Fontanesi et al., 2020; Patrick et al., 2020). For children, COVID-19-related changes in functioning include more symptoms of depression and anxiety, sleep difficulties, and school-related stress (Garcia de Avila et al., 2020; Hussong et al., 2020; Xie et al., 2020; Zhou et al., 2020). Pandemic-related changes in family functioning, however, have been less studied than changes in individual family member’s functioning. Yet, emerging findings suggest that the pandemic has, in some cases, increased marital strain and parental stress leading to harsher parenting and weakened parent–child relationships (Chung et al., 2020a, 2020b; Fontanesi et al., 2020).

In addition, recent scholarship has investigated the ways in which certain types of pandemic stressors are impacting family and individual functioning. Craig and Churchill (2020) found that the quarantine period was associated with increased burden in household labor and that Australian mothers and fathers reported a greater dissatisfaction with how that labor was divided during the pandemic. Moreover, Chung et al. (2020a) found that Singaporean couples with strong support from spouses and employers experienced less marital and parental stress during the pandemic and, in turn, that better work–family balance was associated with lower parenting stress and decreased marital conflict. Thus, emerging studies show that pandemic stress and changes in family functioning are impacting individual functioning. Yet, we know little about how pandemic stress is directly and indirectly impacting family functioning.

## Direct Effects of Pandemic Stress on The Family and Parent–Child Systems

In the current study, our focus on pandemic stress was guided by hypotheses regarding how such stressors impact multiple levels of family functioning and how family functioning, in turn, impacts youth adjustment. Specifically, we indexed family functioning via parent and youth reports of overall family satisfaction, parental support in the parent–child relationship, and openness of family communication. These three dimensions assess aspects of family climate, family processes key to healthy functioning, and the dyadic relationship within the family system, respectively. Moreover, these three indices tap into factors that promote family resilience, as noted by Walsh (2006), that include family cohesion (i.e., family satisfaction and parental support of youth) and coping strategies (i.e., open family communication).

To our knowledge, studies of the current pandemic’s impact on family satisfaction, as a broad indicator of overall family functioning, remain forthcoming. Although studies regarding family communication and COVID-19 are also yet to emerge, commentaries stressing the importance of open communication have appeared. For example, Gambin et al. (2020) suggest that quarantining during the pandemic provides a unique opportunity for families to talk about their emotions. Other commentators agree that parents who communicate with their children in a calm, honest, and direct manner may mitigate the possibility of triggering stress disorders (Dalton et al., 2020; Roccella, 2020). Parent–child relationship quality and support, on the other hand, has been the focus of early empirical studies on family functioning during the pandemic. Notably, Russell et al. (2020) found that child stress was linked with greater parent–child relationship conflict and less closeness. Moreover, these negative effects were worse in families where caregivers reported more distress.

In sum, there is reason to believe that various forms of pandemic stress will impact the family system, including through broad indicators of healthy family climate (family satisfaction) and family process (open communication) as well as more specific indicators of the parent–child system (parent support of youth), which in turn may impact youth functioning. For this reason, we examined whether parent- and child-reported pandemic stressors were associated with changes in each of these three indices of family functioning from before the pandemic to the early months of the pandemic onset.

## Spillover Effects

Relying on prior work by Cummings and colleagues (Kouros et al., 2014; Zemp et al., 2017), Liu and Doan (2020) note that COVID-19-related stressors, such as those related to parents’ work or children’s online schooling, may “spillover” into family life, affecting marital, parent–child, and sibling relationships. Emerging studies indeed show that greater parental stress is related to higher rates of symptomatology during the pandemic for children (Brown et al., 2020; Origlés et al., 2020; Romero et al., 2020; Russell et al., 2020) and that pre-existing child symptomatology predicts greater stress for families during the pandemic (Spinelli et al., 2020). For instance, children whose parents experience greater stress during the pandemic show greater changes in mood and behaviors (Orgilés et al., 2020; Romero et al., 2020) as well as increased stress and parent-child conflict (Russell et al., 2020; Spinelli et al., 2020).

Spillover effects are commonly discussed with respect to parental stress spilling over to impact children via their influence on family functioning (Kouros et al., 2014). For instance, parental stress resulting from the pandemic has been found to be associated with children’s stress and, in turn, parent–child conflict (Russell et al., 2020; Spinelli et al., 2020). Untested for the current pandemic are the types of stressful events that are more likely to result in spillover effects and the aspects of family functioning that are more likely to mediate this effect. In addition, less is known about how child stressors are spilling over into the family, although Russell et al. (2020) found that child stress was linked with greater parent–child relationship conflict and less closeness during the pandemic. Moreover, evidence of greater school-related stressors, illness related concerns, and isolation (Ammar et al., 2020; Saurabh & Ranjan, 2020) have been linked with child distress and symptoms and, as in past quarantine periods, may be traumatic for youth (Sprang & Silman, 2013).

As such, family stressors are expected to have broad effects on family functioning both directly as well as indirectly through spillover effects. Although little research has examined what types of stressors may produce spillover effects, prior work on parental role conflict (i.e., work–family) and caregiver burden may be particularly relevant to COVID-19 life events that predict parental stress and in turn child functioning. In addition, school-related concerns may play a similar role in creating child stress that impacts family functioning and parenting distress.

## Protective Factors

The impact of quarantine practices, involving more social isolation from those outside the family but potentially greater contact with those inside the family home, are posited to have diverging implications for individual and family functioning. Carroll et al. (2020) found that although some parents are struggling, others report that their families are eating healthier, involved in meal preparation, spending dinners together, reporting less stress getting ready for school, and viewing their children as positively adapting to the pandemic. Such “silver linings” may then serve to protect family functioning from the more severe impacts of the pandemic.

In addition, the extent to which parents receive social support from outside the family may safeguard against decrements in family functioning (or in some cases, even enhance family functioning) during the pandemic. For example, Gambin et al. (2020) found that Polish parents’ own social support helped promote positive parent–child relationships during COVID-19 and that such measures can be utilized to promote family closeness during lockdown. Individuals with strong support from spouses and employers and a positive work-life balance during the pandemic also report less marital and parental stress than those with lower levels of support and less balance (Chung et al., 2020a). Similarly, Brown et al. (2020) found that parents who received more support from others reported less perceived stress.

Whereas some commentators suggest that greater time together under pandemic stress may result in greater conflict in some families (Buttell & Ferreira, 2020; Campbell, 2020), others note the potential for greater closeness in some families (Gambin et al., 2020). Such family-level protective factors or positive life event changes during the pandemic; however, these factors have received little attention in the literature despite their potential to inform family programming.

## The Current Study

In the current study, we used data from an existing longitudinal study with assessments occurring before the pandemic began and during the first months of the pandemic onset (May to July, 2020) to assess changes in family functioning as well as associated stressors and youth mental health. In our analyses, we tested six hypotheses. First, we hypothesized that the pandemic was associated with decrements in family functioning (family satisfaction, open communication, marital satisfaction, and parent support of youth; hypothesis 1) in both parent and youth report. Second, we posited that stressors and positive events would predict changes in family functioning from pre-pandemic to during the pandemic (hypothesis 2). Third, we anticipated that pandemic stressors would impact family functioning indirectly via a spillover process. Specifically, negative life events may heighten parental distress and decrease marital satisfaction, which in turn would predict poorer family satisfaction, communication, and parent support of youth (hypothesis 3). Fourth, we expected that the presence of greater social support for parents, however, would forestall increased parental distress and reduced marital satisfaction, indirectly protecting family functioning (hypothesis 4). We postulated that changes in family functioning, resulting from these risk and protective processes (in aggregate), would predict changes in youth mental health symptoms from pre-pandemic to during the pandemic (hypothesis 5). Finally, we explored whether pre-pandemic family functioning predicted experiences of the pandemic. Specifically, we examined which aspects of the family system, parenting sub-system, and parent–child sub-system predicted experiences of pandemic-related stress and positive life events that in turn predicted co-occurring changes in family functioning during the pandemic (hypothesis 6).

## Method

### Participants and Procedures

Participants from the BLINDED study (BLINDED) comprised the sample for this longitudinal analysis. We originally recruited participating parents and children from North Carolina, USA, in 2013–2014 through mass emails to faculty, staff, and students at an affiliated university; flyers distributed through public and independent schools in first- to third-grade classrooms; and community postings. Inclusion criteria were English proficiency and having a child aged 6–9 years.

A total of 101 dyads gave consent/assent to participate at wave 1 in a lab-based data collection and short-term follow-up survey (waves 1 and 2) and 98 participated in a 1.5-year follow-up online parent survey (wave 3). For wave 4 (collected in 2018), a total of 94 participants (90 of the original sample plus four pilot families added in this wave) completed a lab-based data collection followed either immediately or after 1 month by an online parent training program in gratitude socialization. All but seven of the dyads in the analysis sample completed the program in 2018. Wave 9 occurred 3 years later with 90 parents and 88 children from 91 families completing 45-minute online surveys between May 13, 2020 and July 1, 2020. Each received US$25 for participating. All research activities received institutional review board approval.

The analysis sample for the current study included parent–child dyads who participated in either or both data collection efforts before (wave 4) or during (wave 9) the pandemic (*n* = 105 families). These families included 85 families present in both waves, 11 in wave 4 only, and 5 in wave 9 only, necessitating missing data analysis (see below). Sample characteristics include: 48% boys; 80% white, 1% Alaska Native/American Indian, 9% Asian/Asian American, 4% Black/African American, 4% Latinx, and 4% other (summing to over 100% due to individuals identifying with more than one race/ethnicity); 87% mothers; 25% high school graduate without college education, 30% degree from 4-year college, 45% graduate or professional school graduate; and child age at wave 4 = 8 to 13 (*M* = 10.6; *SD = 1.17*) and at wave 9 = 12 to 16 (*M* = 13.6; *SD* = 1.19).

### Measures

Parents reported their child’s *gender* (0 = female) and *age* as well as their own *race/ethnicity* (1 = racial/ethnic minority or multi-racial identity). We standardized and then averaged five items to index *socio-economic status*, including parent report at wave one of (1) approximate family income from the previous year ranging from 0 (US$9999 or less) to 13 (US$200,000 or more); (2 and 3) educational attainment of each parent using an 8-point scale that ranged from 1 (some high school) to 8 (completed graduate or professional degree); and (4 and 5) the MacArthur scale of subjective social status in which parents indicated their own socio-economic status relative to individuals in the United States broadly as well as the socio-economic status of their family of origin (Adler & Stewart, 2007; score *M* = 0; *SD* = .72). Because all but seven of our families completed the online gratitude training program between waves 4 and 9, we also included an indicator of *program participation* as an additional control variable. We examined these five indicators as potential covariates in our analyses.

Table 1 reports all descriptive statistics for the following measures. All scale scores represent the mean of available items, with higher scores reflecting greater presence of the measured construct.

**Table 1.** Correlations and Reliability Estimates among Primary Study Variables.

Variables	1	2	3	4	5	6	7	8	9	10	11	12	13	14	15	16	17	18	19	20	21	22	23	24	25	26
1. Child age	—	—	—	—	—	—	—	—	—	—	—	—	—	—	—	—	—	—	—	—	—	—	—	—	—	—
2. SES	-.19	—	—	—	—	—	—	—	—	—	—	—	—	—	—	—	—	—	—	—	—	—	—	—	—	—
Wave 4 variables																										
3. PR family open comm	-.18	.04	—	—	—	—	—	—	—	—	—	—	—	—	—	—	—	—	—	—	—	—	—	—	—	—
4. CR family open comm	-.15	.20	.13	—	—	—	—	—	—	—	—	—	—	—	—	—	—	—	—	—	—	—	—	—	—	—
5. PR marital satisfaction	-.08	.13	.15	.13	—	—	—	—	—	—	—	—	—	—	—	—	—	—	—	—	—	—	—	—	—	—
6. PR parental support	-.06	-.05	.25	.03	.02	—	—	—	—	—	—	—	—	—	—	—	—	—	—	—	—	—	—	—	—	—
7. CR parental support	-.07	.02	.01	.32	.02	.43	—	—	—	—	—	—	—	—	—	—	—	—	—	—	—	—	—	—	—	—
8. PR family satisfaction	-.14	.05	.65	.11	.38	.40	.22	—	—	—	—	—	—	—	—	—	—	—	—	—	—	—	—	—	—	—
9. CR family satisfaction	-.17	.13	.25	.55	.22	.22	.50	.27	—	—	—	—	—	—	—	—	—	—	—	—	—	—	—	—	—	—
10. PR child symptoms	-.11	.07	-.23	.01	-.32	-.26	.03	-.29	-.20	—	—	—	—	—	—	—	—	—	—	—	—	—	—	—	—	—
11. CR child symptoms	-.01	-.26	-.07	-.18	-.15	-.05	-.10	-.08	-.35	.33	—	—	—	—	—	—	—	—	—	—	—	—	—	—	—	—
Wave 9 variables																										
12. PR family open comm	.07	-.09	.51	-.00	.21	.16	.10	.51	.17	-.25	-.07	—	—	—	—	—	—	—	—	—	—	—	—	—	—	—
13. CR family open comm	-.09	.11	.31	.18	.24	.21	.23	.28	.45	-.26	-.16	.33	—	—	—	—	—	—	—	—	—	—	—	—	—	—
14. PR marital satisfaction	-.03	.09	.11	.03	.72	-.07	.06	.36	.07	-.23	.02	.27	.19	—	—	—	—	—	—	—	—	—	—	—	—	—
15. PR parental support	.04	-.05	.31	.03	.23	.60	.22	.37	.18	-.30	-.16	.54	.33	.17	—	—	—	—	—	—	—	—	—	—	—	—
16. CR parental support	-.13	.18	.24	.21	.28	.36	.45	.29	.38	-.22	-.13	.17	.68	.22	.41	—	—	—	—	—	—	—	—	—	—	—
17. PR family satisfaction	-.01	.01	.28	.06	.35	.17	.28	.48	.23	-.37	.01	.71	.47	.48	.49	.40	—	—	—	—	—	—	—	—	—	—
18. CR family satisfaction	-.15	.12	.29	.31	.33	.20	.34	.25	.46	-.16	-.18	.28	.77	.25	.33	.71	.42	—	—	—	—	—	—	—	—	—
19. PR child symptoms	-.03	-.03	-.22	-.05	-.27	-.33	-.04	-.26	-.18	.64	.30	-.39	-.23	-.16	-.51	-.22	-.39	-.16	—	—	—	—	—	—	—	—
20. CR child symptoms	-.05	-.19	-.19	-.20	-.21	.05	-.12	-.10	-.36	.30	.50	-.23	-.16	.02	-.12	-.40	-.20	-.47	.30	—	—	—	—	—	—	—
21. PR social support	.17	.01	-.02	-.02	.45	-.11	-.04	.17	-.01	-.07	-.08	.25	.09	.52	.19	.13	.27	.04	-.12	-.15	—	—	—	—	—	—
22. PR parent functioning	.18	-.16	.01	-.07	-.27	.12	-.00	-.12	-.20	.13	.11	-.03	-.02	-.16	.01	-.19	.00	-.06	.23	.21	-.07	—	—	—	—	—
23. PR negative stressors	.05	.04	-.06	-.22	-.39	.03	-.12	-.27	-.26	.23	.09	-.18	-.06	-.30	-.05	-.13	-.27	-.19	.22	.07	-.09	.34	—	—	—	—
24. CR negative stressors	.06	.04	-.05	-.07	-.09	.18	.07	.07	-.06	.00	.20	-.07	-.05	.22	.01	.04	-.03	-.12	.07	.40	.04	.01	-.04	—	—	—
25. PR positive events	.04	.03	.11	.14	.10	-.08	.01	.17	.12	.08	.18	.14	.03	.27	.11	.14	.19	.16	.12	.01	.11	-.15	.03	.12	—	—
26. CR positive events	-.07	.32	.22	.13	-.02	-.01	.06	.02	.14	-.10	-.15	.13	.30	-.03	.18	.28	.17	.15	-.15	-.12	-.06	-.02	.07	.00	-.08	—
Mean	10.6	.03	3.97	3.95	5.60	4.18	4.09	3.85	4.04	0.41	0.56	4.05	3.77	5.71	4.12	3.80	3.93	3.84	.56	.73	3.60	.00	.01	.01	.00	.00
Variance	1.35	.53	.23	.31	.80	.25	.33	.24	.33	.06	.07	.31	.35	1.35	.22	.55	.36	.47	.08	.10	.28	.99	.26	.62	.99	.99
Reliability estimate	--	--	.84	.84	.90	.85	.82	.86	.85	.89	.86	.89	.84	.94	.78	.87	.93	.90	.90	.90	.95	.67	—	—	—	—

*Note.* PR = parent report; CR = child report; P-C = parent–child; Comm = communication. Dashed lines indicate reliability estimate not included because internal reliability is not appropriate to life events indices. Correlations greater than .19 are significant at *p* < .05, greater than .25 are significant at *p* < .01, and greater than .32 at *p* < .001.

#### Youth Mental Health

We used the Pediatric Symptom Checklist to assess parent and child report of child symptomatology (Jellinek et al., 1988) at waves four and 9. Respondents indicated how often in the past month the child had exhibited 35 internalizing, externalizing, and attentional symptoms on a scale ranging from 0 (never) to 2 (often). Sample items include “Complained of aches/pains” and “Been fidgety, unable to sit still.” Higher mean scores indicated greater child impairment. For this and each of the following measures, all items were averaged within time points (wave four or 9) and within respondents (parent or child) to obtain composite scores for analysis. Cronbach’s alpha internal reliability estimates for the Pediatric Symptom Checklist were strong at both waves 4 (α = .86, child report; α = .89, parent report) and 9 (α = .90, child report; α = .90, parent report).

#### Parent Social Support of Youth

Parents and youth completed the eight item Arizona Social Support Interview Schedule (ASSIS; Barrera et al., 1981) at waves 4 (using the original past 3-month directions) and 9 (using a past month time frame to capture the pandemic period). At wave 4, children separately rated their relationship with each of two caregivers (typically mother and father) when applicable but rated both relationships together at wave 9. Wave 4 child reports for each caregiver were moderately correlated and averaged to form a single scale score (*r* = .48, *p* < .001). Sample items include “How much can you rely on your parent to really care about you without this changing from time to time” and “How much does your parent give you good advice about how to handle problems that you have?” For both parents and children, item responses ranged from 1 (little or none) to 5 (the most possible). This adapted scale demonstrated satisfactory internal reliability at both waves 4 (α = .82, child report; α = .85, parent report) and 9 (α = .87, child report; α = .78, parent report).

#### Parent’s Marital Satisfaction

The respondent’s marital satisfaction was assessed using the seven-item Relationship Assessment Scale (Hendrick, 1988) by parent report at wave 4 (with the original directions not indicating a time frame) and 9 (using a past month time frame). For the first four items of the scale, participants responded to items assessing relationship satisfaction with responses ranging from 1 (very low satisfaction) to 7 (very high satisfaction). Next, participants indicated how often they regretted the relationship (reverse scored) and how much they loved their partner on two items ranging from 1 (not at all) to 7 (a great deal). Finally, on a single reverse-scored item, participants reported how many problems existed in their relationship with responses ranging from 1 (none) to 7 (very many). Cronbach’s alpha estimates were high at both waves 4 (α = .90) and 9 (α = .94).

#### Family Satisfaction

Both children and parents reported on family satisfaction using the Family Satisfaction Scale (FSS; Barnes & Olson, 1985). Reporters indicated the extent to which they agreed with ten items with responses ranging from 1 (strongly disagree) to 5 (strongly agree) using the original directions without a time frame in wave four and in the past month in wave 9. The measure showed strong internal reliability at both waves 4 (α = .85, child report; α = .86, parent report) and 9 (α = .90, child report; α = .93, parent report).

#### Family Open Communication

Parents and children completed the 10-item Family Communication Scale (FCS; Olson & Barnes, 2010). Participants rated agreement with responses ranging from 1 (strongly disagree) to 5 (strongly agree) using the original directions without a time frame in wave four and in the past month in wave 9. Cronbach’s alpha internal reliability estimates for the scale were strong at both waves 4 (α = .84, child report; α = .84, parent report) and 9 (α = .84, child report; α = .89, parent report).

#### Parent Social Support Scale

At wave 9, parents completed the abbreviated version of the social support scale from the Support Provision Scale (Cutrona & Russell, 1987; Iapichino et al., 2016). Participants rated ten items assessing how well statements described emotional and social support derived from relationships with others in the past month (e.g., family members, friends, or coworkers) using a scale ranging from 1 (strongly agree) to 4 (strongly disagree). Sample items include “There are people who admire my talents and abilities” and “There are people I can depend on to help me if I really need it” (α = .95).

#### Child COVID-19-Negative and Positive Life Events

Children indicated whether they experienced each of 27 COVID-19-related life events and, for those experienced, rated how desirable they found each to be on a scale of −4 (extremely bad) to 0 (neither good nor bad) to 4 (extremely good). The scale included 11 items from the Responses to Stress Questionnaire-COVID-19, an adaptation of the original Responses to Stress Questionnaire for COVID-19 (Compas, 2020; Connor-Smith et al., 2000), and 16 items written for this study (3 negative events and 11 positive items as well as two items without a clear valence, that were dropped for this analysis). The eleven total positive life event items were rescored to range from 0 (collapsing over extremely bad to neither good nor bad) to 4 (extremely good) and assessed, for example, more time outside, finding ways to help people, and more time for hobbies. Similarly, the fourteen total negative life events were rescored to range from 0 (collapsing over extremely good to neither good nor bad) to 4 (extremely bad) and assessed, for example, school stress, illness concerns, and isolation.

#### Parent Pandemic Life Events and Distress

Parents completed the Epidemic-Pandemic Impacts Inventory (Grasso et al., 2020) at wave 9. Parents rated whether they had experienced each of 92 COVID-19-related events. For purposes of this study, we dropped items that directly overlapped with changes in family functioning and created subscales that captured seven dimensions: positive life events (19 items; e.g., “found greater meaning in work” or “volunteered time to help people in need”), work/school stress (four items; e.g., “increased workload”), economic/resource loss (five items; e.g., “laid off from job”), isolation/separation (15 items; “isolated or quarantined due to possible exposure to this disease”), illness concerns (four items; e.g., “had symptoms of this disease but never tested”), caregiving stress (nine items; e.g., “childcare or babysitting unavailable when needed”), and medical provider stress (four items; e.g., “provided direct care to people with the disease”). Scales assessing economic resource/loss, COVID-19 exposure risk, and medical provider stress were scored dichotomously due to lower base rates. Other scales were scored as the proportion of items endorsed. An overall negative stress scale was formed by averaging standardized negative scale scores.

Twelve additional items on this scale indexed changes in parental functioning related to COVID-19, each rated as occurring or not and then averaged to form this scale. Items assessed, for example, changes in mental health, sleep, physical activity, and need for medical care. Such items thus function as a stress impact indicator (though with modest reliability, α = .67).

### Analytic Plan

**Figure 2.**
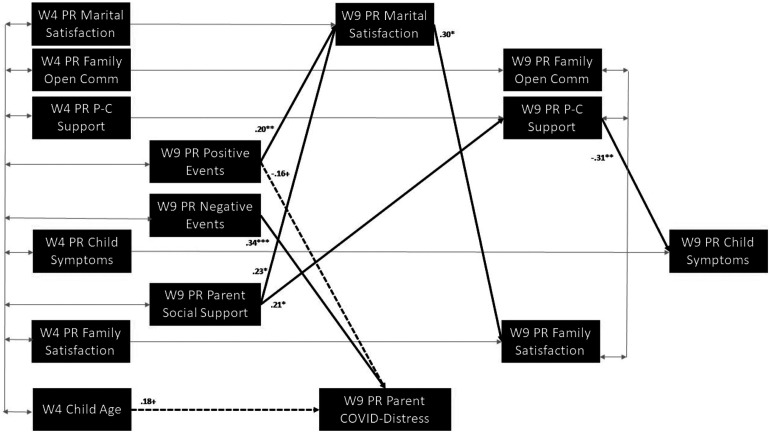
Parent Report Model for Direct and Spillover Effects. *Note.*: PR = parent report; Comm = communication; Qlty = quality. W4 = wave four; W9 = wave 9. Prediction of family open communication and parental support of youth from family satisfaction indicated by boxed A and B pathways, respectively. Gray lines are control pathways. Dark lines are significant at *p* < .05 and dashed lines at *p* < .10.

**Figure 3.**
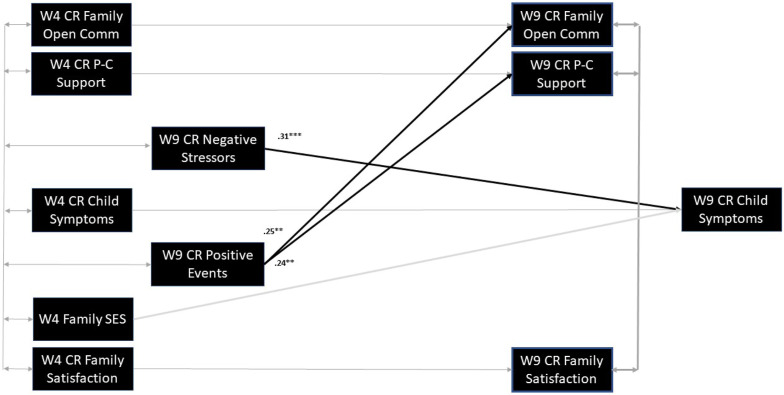
Child Report Model for Direct and Spillover Effects. *Note.* CR = child report; Comm = communication; Qlty = quality; SES = socio-economic status. W4 = wave four; W9 = wave 9. Prediction of family open communication and parental support of youth from family satisfaction indicated by boxed A and B pathways, respectively. Gray lines are control pathways. Dark lines are significant at *p* < .05 and dashed lines at *p* < .10.

To test changes in family functioning by parent and child report from pre-pandemic (wave 4) to during the pandemic (wave 9), we conducted a series of within-person t-tests for family functioning indicators (see Table 2; hypothesis 1). We simultaneously tested direct (hypothesis 2) and indirect (hypothesis 3) effects of pandemic-related negative and positive life events on changes in family functioning as well as the potential protective effects of parents’ social support (hypothesis 4), with implications for changes in youth mental health symptoms (hypothesis 5). We tested theses hypotheses separately for parent and youth report on these variables. To account for missing data (seven children and eight parents in wave 9 were missing wave 4 and 12 children and parents in wave four were missing in wave 9), we generated and synthesized 100 imputations using Mplus (Muthén & Muthén, 1998–2019). Prior to testing hypotheses, we evaluated a covariate-only path model by regressing parent and self-report of youth mental health symptoms (in separate models) on potential covariates at wave 4 (parent race/ethnicity, child gender, child age, family socio-economic status, and prior program participation) using robust maximum likelihood estimation. We then trimmed non-significant pathways (using a conservative *p* < .10) and added predicted pathways (see Figure 2 for parent report model and Figure 3 for child report model).

**Table 2.** Changes in Parent and Child Report of Family Functioning.

Outcome	Sample size	Mean W4	Mean W9	T-test	Cohen’s *d*
Child-reported variables					
Open family communication	89	3.94	3.77	2.12*	.29
Parental support of child	89	4.09	3.80	3.72***	.43
Family satisfaction	89	4.04	3.84	2.84**	.31
Parent-reported variables					
Open family communication	89	3.97	4.05	−0.92	.16
Parental support of youth	89	4.18	4.12	1.82+	.13
Family satisfaction	89	3.84	3.93	−0.56	.15
Marital satisfaction	76	5.60	5.71	−0.93	.11

*Note.* ***, **, *, and + indicate *p* < .001, *p* < .01, *p* < .05, and *p* < .10, respectively. W4 = wave four; W9 = wave 9.

In these models, we predicted residualized change in family functioning variables and in youth mental health by regressing wave 9 indicators of these variables on wave 4 (pre-pandemic) levels of these variables along with other predictors. Residualized change score models allow interpretation of the outcome as a change score when those in the sample are believed to represent a single population (Castro-Schilo & Grimm, 2018). Thus, in Figure 2 (the parent report model results) positive events, negative stressors, parent social support, and parents’ distress during the pandemic serve as predictors of changes (from pre-pandemic to during the pandemic) in the four family functioning indicators (open family communication, parental support of youth, family satisfaction, and parent report marital satisfaction) as well as youth symptoms. To estimate spillover effects, positive events, negative stressors, and parent support served as predictors of parent distress (all assessed during the pandemic), which in turn predicted changes in marital satisfaction. Changes in marital distress in turn predicted changes in other indicators of family functioning. We estimated a parallel model (without parent-reported social support, distress, and marital satisfaction) for child report (see Figure 3 for results).

**Figure 4.**
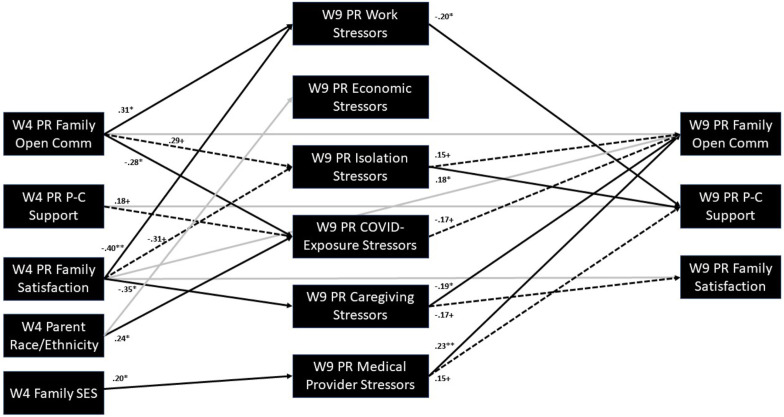
Parent Report Model for Pre-Existing Family Functioning and Pandemic-Related Life Events. *Note.*: PR = parent report; Comm = communication; Qlty = quality; SES = socio-economic status. W4 = wave four; W9 = wave 9. Direct paths from wave four to wave nine family functioning variables depicted by grayed lines (results not reported here). Gray lines are control pathways. Dark lines are significant at *p* < .05 and dashed lines at *p* < .10.

**Figure 5.**
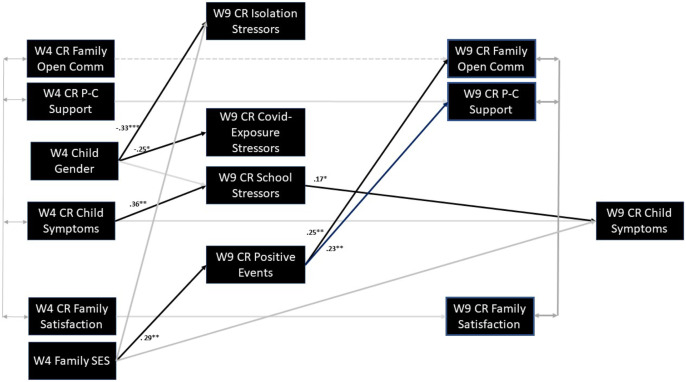
Child Report Model for Pre-Existing Family Functioning and Pandemic-Related Life Events. *Note.* CR = child report; Comm = communication; Qlty = quality. W4 = wave four; W9 = wave 9. Direct paths from wave four to wave nine family functioning variables depicted by grayed lines (results not reported here). Gray lines are control pathways. Dark lines are significant at *p* < .05 and dashed lines at *p* < .10.

To examine how pre-pandemic family functioning predicted experiences of specific types of stressors during the pandemic and in turn residualized change in family functioning indicators, we estimated separate path models by reporter. Once again, we estimated a covariate-only model to determine retained control variables (shown in Figure 4 for parent sreport and 5 for child report models, respectively). Then, we estimated a parent report model using the three indicators of family functioning at wave 4 as predictors of parent-reported stressors (work, economic, isolation, illness concerns, caregiving, and medical provider) which in turn predicted residualized change in the three indicators of family functioning at wave 9 (Figure 4). A parallel child report model included the negative stressors of isolation, illness concerns, and school-related stressors with child reports of the three indicators of family functioning (Figure 5).

Finally, to examine cross-reporter effects, we estimated four additional models. These models tested whether the parent report variables predicted residualized change in youth-reported symptomatology (by re-estimating models in Figure 2 and 4, using child instead of parent reports of youth outcomes) and whether child-reported variables predicted residualized change in parent reports of youth symptoms (by re-estimating models in Figure 3 and 5, using child instead of parent reports of youth outcomes).

Following (Hancock and French, 2013), we estimated power for a single path in our most complex path analysis (depicted in Figure 2) using an analogous 14 predictor regression. Using G-power, we find that with *n* = 105 and α = .05 in a two-tailed test, we have power of β = .80 to detect a small to medium effect (*f*^*2*^*=* .08) using Cohen’s nomenclature.

## Results

### Hypothesis 1: Changes in Family Functioning Pre-Pandemic to During the Pandemic

As reported in Table 2, within-person t-tests showed that only child report variables demonstrated significant change over this time; decreases were noted in family open communication, parental support of youth, and family satisfaction. Note that wave 9 child–reported variables were not significantly correlated with child age.

### Hypothesis 2-5: Parent Report Direct Effects and Spillover Model

Results of the covariate model found only one effect: COVID-19-related changes in parent functioning were greater when the target child was older (*b* = .18; *t* = 1.98; *p* < .05; see Figure 2). We retained this effect in the full model which provided an acceptable fit to the data (*χ*^2^ (32) = 53.18, *p* < .05; RMSEA = .08; CFI = .95; and SRM = .05). Negative and positive life events were not directly related to residualized change in family satisfaction, communication, or social support. However, there were positive spillover effects such that greater positive life events predicted greater marital satisfaction (*b* = .20; *t* = 2.61; *p* < .01) as did greater social support for parents (*b* = .23; *t* = 2.24; *p* < .05). Marital satisfaction was in turn associated with greater parental family satisfaction (*b* = .30; *t* = 2.22; *p* < .05). Greater parent social support also predicted increased (or fewer decrements in) parental support of youth during the pandemic (*b* = .21; *t* = 1.93; *p* < .05), which in turn predicted less child symptomatology (*b* = −.31; *t* = −3.08; *p* < .01; hypothesis 5). Although not associated with family functioning, more impairment in parental functioning associated with COVID-19 was associated with parents reporting more negative COVID-19-related stressors (*b* = .34; *t* = 3.47, *p* < .001), and (although only marginally) fewer positive life events (*b* = −.16; *t* = −1.76, *p* = .08) and having older children in the study (*b* = .18, *t* = −1.82, *p* = .07). The resulting model accounted for a significant proportion of the variance in wave nine family functioning (*R*^2^ = .36, .45, .37, and .63 for open communication, parent support, family satisfaction, and marital satisfaction, respectively) and child symptoms (*R*^2^ = .52).

### Hypothesis 2 and 5: Child Report Direct Effects and Spillover Model

Results of the covariate model showed no significant associations. We thus estimated the child report model without covariates and found an excellent fit to the data (*χ*^2^ (13) = 14.33, *p* > .05; RMSEA = .03; CFI = .99; and SRM = .03). Results showed that negative life events did not predict residualized change in family functioning, but they did predict greater elevation in child symptoms from pre-pandemic to during the pandemic (*b* = .31; *t* = 4.55, *p* < .001 see Figure 3). In addition, experiencing positive COVID-19-related life events was related to fewer decrements in open family communication (*b* = .25; *t* = 2.72, *p* < .01) and parental support of youth (*b* = .24; t = 2.84, *p* < .01) from before to during the pandemic. The resulting model accounted for a significant proportion of the variance in wave nine family functioning (*R*^2^ = .28, .27, and .23 for open communication, parent support, and family satisfaction, respectively) and child symptoms (*R*^2^ = .50).

To examine potential impact of Type 1 error, we performed a Benjamini–Hochberg correction (Benjanimi & Hochberg, 1995) for results reported in Figures 2–5. Using a 20% false detection rate and clustering the number of “repeated tests” as the predictors of wave nine outcomes in a model beyond the control variables, we found only one effect that fell from significance to marginal significance in results reported in Figure 3 (positive child life events predicting child reports of open family communication) and no changes for results reported in Figures 1, 4, or 5.

### Hypothesis 6: Parent Report Model of Family Functioning Predicting Pandemic Stress

Covariate analyses showed greater illness concerns for parents who identified as a racial/ethnic minority (*b* = .38; *t* = 3.25, *p* < .001) and greater medical provider stress for families with higher socio-economic status (*b* = .22; *t* = 2.41, *p* < .05). The resulting model provided an acceptable fit to the data (*χ*^2^ (19) = 30.17, *p* > .05; RMSEA = .08; CFI = .96; and SRM = .04). Decreased family communication was related to more parent caregiving stress (*b* = −.19; *t* = −2.20, *p* < .05) and, although marginally significant, more illness concerns (*b* = −.17; *t* = −1.83, *p* = .07). However, medical provider stress (*b* = .23; *t* = 2.83, *p* < .01) was related to increases (or fewer decrements) in family communication as was greater isolation stress, although this was only marginally significant (*b* = 15; *t* = 1.53, *p* = .10). A decrease in parental support of youth was related to greater work stress (*b* = −.20; *t* = 2.25, *p* < .05), although, like family functioning, greater isolation stress (*b* = .18; *t* = 2.09, *p* < .05) and (at marginal significance) medical provider stress (*b* = .15; *t* = 1.90, *p* = .06) were related to increases (or fewer decrements) in parental support of youth. Greater caregiving stress showed a marginally significant association with decreases in family satisfaction (*b* = −.17; *t* = −1.71, *p* = .09).

In addition, family functioning impacted the likelihood of experiencing pandemic-related stressors. Greater open family communication pre-pandemic predicted more work stress (*b* = .31; *t* = 2.41, *p* < .05) and (at marginal significance) isolation stress (*b* = .29; *t* = 1.84, *p* = .07) as well as fewer illness concerns (*b* = −.28; *t* = −2.21, *p* < .05) during the pandemic. Stronger parental support of youth predicted greater risk for illness concerns, although at marginal significance (*b* = .19; *t* = 18.6, *p* = .06). Greater family satisfaction pre-pandemic predicted less work stress (*b* = −.40; *t* = −3.14, *p* < .01), caregiving stress (*b* = −.35; *t* = −2.30, *p* < .05), and (at marginal significance) isolation stress (*b* = −.31; *t* = −1.80, *p* = .07) during the pandemic. And racial/ethnic minority status predicted greater illness concerns (*b* = .25; *t* = 2.24, *p* < .05) and higher socio-economic status predicted greater medical provider stress (*b* = .20; *t* = 2.03, *p* < .05). The resulting model accounted for a significant proportion of the variance in wave nine family functioning (*R*^2^ = .43, .49, and .31 for open communication, parent support, and family satisfaction, respectively).

### Hypothesis 6: Child Report Model of Family Functioning Predicting Pandemic Stress

Covariate models showed that girls reported greater stress during the pandemic due to isolation (*b* = −.57; *t* = −3.03, *p* < .01), illness concerns (*b* = −.56; *t* = −2.81, *p* < .01), and school-related stress (*b* = −.38; *t* = −1.94, *p* = .05). Higher family socio-economic status predicted less isolation stress (*b* = .31; *t* = 2.21, *p* < .05), greater positive events during COVID-19 (*b* = .44; t = 3.28, *p* < .001), and lower child symptomatology during the pandemic (*b* = −.08; *t* = −1.93, *p* = .05). Controlling for these effects, the model provided an excellent fit to the data (*χ*^2^ (20) = 23.91, *p* > .05; RMSEA = .04; CFI = .99; and SRM = .03; see Figure 5). Results showed that children with more symptomatology pre-pandemic had more school-related stress during the pandemic (*b* = .36; *t* = 3.28, *p* < .001), which predicted greater increases (residualized change) in child symptoms during the pandemic (*b* = .17; *t* = 1.96, *p* < .05). Positive events continued to predict greater open family communication (*b* = .25; *t* = 2.62, *p* < .01) and greater parental support of youth (*b* = .23; *t* = 2.75, *p* < .01). Girls continued to report more isolation (*b* = −.33; *t* = −3.63, *p* < .001) and illness concerns (*b* = −.25; *t* = −.47, *p* < .05) during the pandemic and higher socio-economic status predicted more positive events (*b* = .23; *t* = 2.75, *p* < .01). The resulting model accounted for a significant proportion of the variance in wave nine family functioning (*R*^2^ = .29, .29, and .23 for open communication, parent support, and family satisfaction, respectively).

### Testing Cross-Reporter Effects

We estimated whether parent-reported variables predicted youth-reported symptoms, and vice versa, by re-estimating model shown in Figures 2 and 3. We found no cross-reporter effects predicting youth symptoms in either model, although in both cases model fit was acceptable (*χ*^2^ (32) = 49.98, *p* < .05; RMSEA = .07; CFI = .95; and SRM = .04 for parent-reported predictors and *χ*^2^ (13) = 17.49, *p* > .05; RMSEA = .05; CFI = .98; and SRMR = .04 for child-reported predictors).

Similarly, we estimated whether parent-reported family functioning was associated with child-reported stressors and vice versa, by re-estimating models shown in Figures 4 and 5. In the first, the parent reports of family functioning model fit the data well (*χ*^2^ (13) = 19.91, *p* > .05; RMSEA = .07; CFI = .98; and SRM = .04). Greater child-reported school stress predicted decrements in parent reports of parental support of youth (*b* = −.10; *t* = −2.35, *p* < .05), although no other effects of child-reported stressors on changes in parent-reported family functioning were found. Higher parent-reported parental support of youth pre-pandemic also predicted greater isolation stress by child-reported during the pandemic (*b* = .30; *t* = 2.55, *p* < .05). In addition, lower parent-reported open family communication (*b* = −.27; *t* = −2.22, *p* < .05) and, unexpectedly, higher parent-reported family satisfaction (*b* = .31; *t* = 2.20, *p* < .05) predicted greater child-reported school stress during the pandemic.

The second model of child-reported family functioning and parent-reported stressors also showed acceptable fit (*χ*^2^ (27) = 47.79, *p* < .05; RMSEA = .09; CFI = .92; and SRM = .07). Parent-reported medical stressors predicted greater child-reported family open communication (*b* = .22; *t* = 2.34, *p* < .05), positive parental support of youth (*b* = .18; *t* = 2.16, *p* < .05), and (although only marginally significant) higher family satisfaction (*b* = .15; *t* = 1.65, *p* = .10). In addition, greater open family communication pre-pandemic predicted less caregiving stressors by parent report during the pandemic (*b* = .-21; *t* = −1.98, *p* < .05) and a marginally significant effect was found for child-reported family satisfaction to lower medical provider stress during the pandemic by parent report (*b* = −.25; *t* = −1.75, *p* = .08).

## Discussion

The current longitudinal study examined changes in family functioning in the southeastern United States from pre-pandemic into the early months of the pandemic, pandemic-related processes that both impair and safeguard functioning, and implications of changes in family functioning for child symptomatology. According to child report of family functioning, open family communication, parental support of youth, and family satisfaction all decreased during this time, although no changes were found in parent report of family functioning. Several forms of parent-reported negative life events and child reports of school-related stress during the pandemic predicted changes in family functioning. Moreover, positive life events predicted child reports of family functioning directly and evidence was found for spillover effects of parent-reported positive life events on family functioning. In addition, the receipt of social support by parents during the pandemic protected against decrements in family functioning and, indirectly, increases in child symptomatology. School-related stress, on the other hand, predicted increases in child-reported symptomatology. Finally, several aspects of family functioning pre-pandemic impacted the extent to which parents and children experienced both positive and negative life events during the pandemic. Thus, the current findings shed light on how experiences of the pandemic are linked with family functioning.

The importance of examining multiple reporters in understanding family processes is once again evident in the current study, most notably in the consistent decrements in family functioning that were found across indicators for child but not parent report from pre-pandemic to the first months of the pandemic quarantine period. Such differences may be due to several factors. For example, parents may adjust their expectations and ratings for family functioning during the pandemic, whereas children may not include such allowances for context. In addition, younger teens may find the increased time with family confining, restricting their goals for autonomy and independence, and, as a result, experience family functioning more negatively than do parents.

Although the current findings cannot distinguish these differences, results do provide insights into factors that may improve or protect against pandemic-related decrements in family functioning as well as consequences of such decrements. First, child-reported positive events (including spending time with people, being active, spending time outside, and engaging in hobbies and creative activities) were associated with more open family communication and stronger parental support of children during the pandemic. Second, parental social support both directly protected parental support of youth during the pandemic and indirectly increased family satisfaction by increasing marital satisfaction. This finding underscores that spillover effects, including those related to parents receiving social support, may not only improve family functioning by their impact on marital relationships but also protect children against pandemic-related increases in symptomatology by reinforcing the quality of the parent–child relationship. And, third, parents reporting greater isolation in the early months of the pandemic quarantine also reported a more positive parent–child relationship, perhaps due to welcome family togetherness, although child reports showed no such effect. In general, these findings suggest that in the face of stressors associated with the COVID-19 pandemic, remaining positively engaged with others, seeking and receiving social support, and remaining physically and mentally engaged in activities may all be important ways to improve individual health (as previously suggested, Killgore et al., 2020) and family functioning.

A somewhat surprising finding was that stress associated with having a parent actively engaged in providing medical care for those impacted by COVID-19 was also protective against some decrements in family communication during the pandemic, by both parent and child report. In the early months of the pandemic quarantine in particular, such families may have been privy to more accurate information about the pandemic and have talked more openly about this information. Families may rally around parents in frontline jobs and such parents may especially value family support and their relationships with children as they face the daily realities of the pandemic at work. Thus, although such care providers clearly have experienced significant stress and demands in their work that impact them in other ways (Craig & Churchill, 2020), for some these experiences may bring their family closer or at least protect against pandemic-related decrements in family functioning during this time.

Findings for this study also affirm some of the sources of stress that appear to have impaired family functioning as the pandemic quarantine unfolded. For youth, none of the negative life event domains (isolation, illness concerns, and school) predicted changes in family functioning, although greater school stress predicted increased symptomatology. Pandemic-related work and caregiving stressors were most impactful for parents, with work stress associated with decreased parental support of youth and caregiving stress with less open family communication. These stressors underscore the extreme challenges to family–work balance that parents are facing and that impact families as a whole (Craig & Churchill, 2020).

In terms of how these processes impact youth, we saw limited direct effect of pandemic-related life events on child symptomatology although school-related stressors increased child-reported symptomatology. On the other hand, greater parental support of youth protected against increased symptomology. However, as rising trends in youth mental health problems have begun to emerge as the pandemic and corresponding safety measures continue (e.g., Garcia de Avila et al., 2020; Jiao et al., 2020; Xie et al., 2020), changes in how these family processes impact youth functioning are likely to emerge as well. For example, it is important to note that decisions about Fall 2020 in-person school closures and the move to digital learning that occurred in this area had yet to be announced at the time of this data collection.

In addition, findings from this study suggest that aspects of family functioning prior to the pandemic may serve to protect against or leave family members vulnerable to pandemic-related stressors. Most notably, pre-pandemic (parent-reported) family satisfaction and (child report) open family communication resulted in less parental stress (work and caregiving) during the pandemic. Open family communication as reported by parents also resulted in fewer illness concerns, perhaps by providing ready skills for discussing the uncertainties and challenges of the pandemic as well as illness stressors within the family.

We also found that, as compared to boys, girls were more vulnerable to isolation stress and, along with racial/ethnic minority families, illness concerns. Finally, families with a relatively higher socio-economic status reported greater medical provider stress and child reports of positive life events. These findings parallelly reported disparities in COVID-19 exposures and impact in the literature, with greater negative stress for racial/ethnic minority families and more protective factors for families with greater socio-economic resources (Abedi et al., 2020) even within this relatively privileged sample.

### Limitations

Strengths of this study include the use of a longitudinal design, the use of multiple reporters, and the consideration of various domains of life events that can pose both risk and protective effects on families coping with the pandemic. Limitations include the presence of only two time points, inclusion of a single parental perspective (often mothers) and no siblings, and restricted ethnic and racial diversity in the sample. This last point is particularly important given that the pandemic has disproportionately impacted racial/ethnic minority families and families with low socio-economic resources (Abedi et al., 2020). Understanding how the pandemic has impacted a wide range of families, both within the United States and across the globe, will be critical in supporting the developmental recovery of youth and their families in the years to come. Thus, the current study offers a snapshot of how the pandemic has impacted a relatively privileged sample as one of many needed contributions to this research. Last, participants were drawn from a sample already engaged in a study of gratitude intervention, offering some opportunity for a self-selection bias. These participants may have had a more distinct focus on family functioning and positive emotion, which may be reflected in the findings of the current study.

As future research continues to examine these questions, these findings underscore the importance of longitudinal data that can address temporal precedence and developmental progression as well as multiple reporter data, given differences in perspectives (and the potential for reporter bias) particularly in family research. Indeed, the most salient finding across reporters was the protective effects on family functioning of having a frontline medical provider as a parent. This finding suggests that families who experience pandemic-related stress through an exposed caretaker may be finding ways of engaging in family processes that are adaptive to this unique situation, perhaps identifying approaches that may be useful for families who experience related stressors. Further research into the family-level coping strategies of this subgroup could thus be useful for service planning. Additionally, there is great heterogeneity in individual and family responses to the COVID-19 pandemic, its seriousness, and its related safety precautions. Further investigation of these varying views and beliefs about the pandemic may offer deeper insight into how families may be differentially experiencing pandemic-related stressors and aspects of family functioning.

These findings have other implications for supporting families during the COVID-19 pandemic and related crises. First, as reported elsewhere, there are predictable differences in the stressful life events to which families may be exposed. Families with fewer resources and those who may be marginalized due to race/ethnicity are experiencing more negative illness–related impacts of the pandemic and fewer of the positive life events, which may require more time and money to access. Some youth, in the current study girls more so than boys, may also be more susceptible to the social changes of the pandemic that include more isolation. Understanding the long-term impact of such isolation on youth development and the role of families in mitigating that risk is an important research agenda, just as finding ways to help youth stay connected is a clear service implication of these results.

Second, helping families, not only youth, stay connected to others who provide support is important for parent, family, and child functioning. The closure of workspaces, leisure spaces, and community spaces (public parks and places of worship) has the potential to limit the amount of social support and contact that parents have as they navigate their lives during the pandemic. Seeking social support under quarantine and social distancing practices likely requires more active, intentioned behavior from parents who may feel squeezed for time. Encouraging and facilitating social support seeking for parents, however, is not simply a matter of self-care but also of family care.

Third, these findings also suggest that helping families maintain positive parent–child relationships and reducing school stress, sometimes competing goals when parents become teachers and enforcers for online learning, are both important avenues for reducing increases in symptomatology in children. Integrating support through schools, community organizations, and mental health providers, particularly for vulnerable families with children already evidencing symptomatology prior to the pandemic, is one avenue forward in meeting this need.

And, finally, positive events for children and parents are another avenue for supporting family functioning. These events include spending quality time together, taking care of the physical needs of family members (e.g., diet, sleep, and exercise), and engaging in enjoyable activities. These may well be the very activities that are most difficult for families to maintain under the conflicting work and caregiving demands of the pandemic. Finding ways to support families in making time for these activities, without adding to parental stress or a sense of failure for not doing them, is another way to enhance family functioning during this time.
